# Quantum Chemical Calculations on the Interaction between Flavonol and Functional Monomers (Methacrylic Acid and 4-Vinylpyridine) in Molecularly Imprinted Polymers

**DOI:** 10.3390/molecules15064017

**Published:** 2010-06-02

**Authors:** Luis Enrique Gómez-Pineda, Georgina Esther Pina-Luis, Carlos Martín Cortés-Romero, Manuel Eduardo Palomar-Pardavé, Giselle Alicia Rosquete-Pina, Marta Elena Díaz-García, María de los Angeles Cuán Hernández

**Affiliations:** 1 Graduate Center & Research, Technological Institute of Tijuana, P.O. 1166, Tijuana, B. C. 22500, Mexico; 2 IATC, 76802 San Juan del Río, Querétaro, Mexico; 3 Materials Department, Metropolitan Autonomous University-Azcapotzalco, Av. San Pablo 180, Col. Reynosa Tamaulipas, 02200 México D.F., Mexico; 4 Department of Physical and Analytical Chemistry, Faculty of Chemistry, University of Oviedo, Av. Julián Clavería 8, 33006 Oviedo, Spain

**Keywords:** DFT study, pre-polymerization complex, imprinted polymer

## Abstract

Quantum chemical calculations were performed to characterize the interaction of the flavonol molecule (FL) with methacrylic acid (MAA) and 4-vinylpyridine (4VPy) in the formation of imprinted polymers. The polarizable continuum model (PCM) was used to gain insight on the type of interaction between the reactant molecules under vacuum conditions and in the presence of different solvents. The effect of solvent on the pre-polymerization complex formation was evaluated through the stability energy, in which chloroform behaves as the best solvent for the synthesis of the imprinted polymers since it facilitates the reaction by lowering its degree of stabilization. The reactivity was analyzed in terms of the electrostatic surface potential (ESP) and Mulliken charge. By means of these results, it has been possible to determine two potential recognition sites for the interaction of the MAA monomer and one for the 4VPy in relation to the strength of interaction with FL. In this concern, the interaction of the system FL-MAA is stronger than FL-4VPy.

## 1. Introduction

The molecular imprinting technique is a specific chemical procedure for the generation of explicit nano-cavities that are capable of recognizing and binding a desired molecular target with high affinity and selectivity. Although the technology of molecular imprinting has been widely used for polymer synthesis, very few investigations have been performed to understand the factors that drive the imprinting process with the aim of predicting molecularly imprinted polymer (MIP) performance. Self-assembly molecular imprinting relies on the nature and stability of the complex formed between the target analyte and functional monomer building block prior to radical polymerization, which ultimately governs the amount of binding sites with high selectivity and specificity [1,2,3,4,5,6,7,8,9].

The rational optimization and design of a MIP requires fundamental information concerning its structure and function under ideal conditions. An alternative approach for the study of the complex is based on molecular modeling, since it provides fundamental knowledge. Theoretical chemistry can give an insight into some of the issues since it can determine the structure of the MIP, the active sites, and atomic level description of interaction mechanisms on the complex formation involved in a given reaction [10,11,12,13].

The use of a combined theoretical and experimental approach represents an evident advantage, since it increases confidence in the results and is an important verification of the accuracy of both methods [10,11,12,13]. In the present paper, the interaction of the flavonol molecule (FL) with the functional monomers methacrylic acid (MAA) and 4-vinylpyridine (4VPy) in imprinted polymer formation have been studied theoretically. Density functional theory method at B3LYP/6-31+G(d,p) was employed. The influence of different solvents in the pre-polymerization complex formation was also evaluated. ^13^C-NMR was used to confirm the presence of interactions between functional monomers and flavonol. Flavonol was chosen as a representative target compound because it is a typical member of the large group of phytochemicals ubiquitously found in fruits and vegetables, which are well known for their antioxidant abilities and hold promise for preventing age-related diseases including heart disease and cancer [14,15,16]. On the other hand, flavonol containing hydroxyl and carbonyl groups for intermolecular interactions with the functional monomers during self-assembly imprinting [17].

## 2. Results and Discussion

### 2.1. Conformational analysis

**Figure 1:**
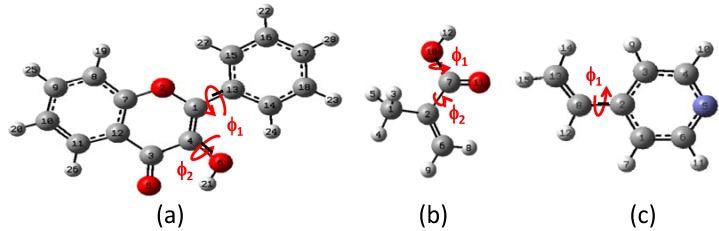
Torsion angles ф_1_ and ф_2_. (a) Flavonol (FL), (b) methacrylic acid (MAA) and (c) 4-vinylpyridine (4VPy).

**Figure 2:**
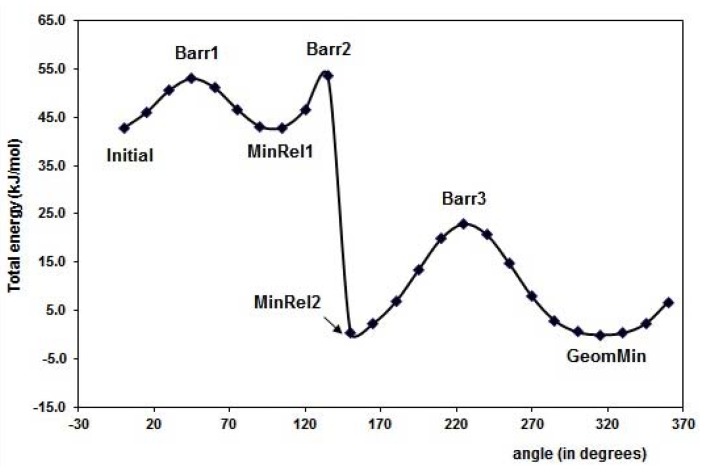
Graphical representation of the PES for flavonol molecule, for the dihedral angles torsion (ф_1_ and ф_2_), see Figure 1 (a). The corresponding selected geometry structure is in Table 1.

The results for optimal energy minimization conformation were obtained using DFT at B3LYP/6-31+G(d,p) level of theory. The search for the minimum of the simple molecules of FL, MAA and 4VPy was able to be performed by the torsion of the dihedral angles ф_1_ and ф_2_ as it is depicted in Figure 1. The schematic representation for FL is shown in Figure 2 making use of the potential energy surfaces (PES) which is split in two parts mainly. The first located in the upper part is related with ф_2_ rotational angle, where the hydrogen (H21) of the –OH group is placed in the contrary direction to -C=O group and the lower part is about the ф_1_ rotational angle where the hydrogen (H21) from the –OH group is pointed out to the oxygen O5 from the –C=O group. The corresponding geometries of the minimum and maximum along the PES are summarized in Table 1.

The minimal energy conformation corresponds to a nearly planar conformation of the molecule, which is in line with previous theoretical studies reported [18]. Experimental techniques proved a twisted favorable conformation of flavones (ф = 28.3°) in vapor phase [19], in contrast with the crystal phase, where the molecule is nearly planar [20]. Our results show that the hydrogen H21, from the –OH, is pointed out to the -C=O group, giving this orientation 43 kJ mol^-1^ of energy stability to the flavonol molecule with respect to the MinRel1 conformation. It is worth to mention that MinRel2 conformation and the minimal energy conformation have the same energy value. The GeomMin gives a planar conformation, while the MinRel2 gives nearly planar conformation (ф_1_ = 13.35 grades) and the intermolecular distance between O5 and H21 is of 1.98 Å for both, rather close to the crystal phase [20]. Becke’s B3LYP hybrid functional used for the DFT calculations contains a combined exchange functional and combined correlation functional [21,22] and is widely used because it leads to reliable results.

**Table 1:** The corresponding geometries of the graphical representation in the Figure 2, Figure 3 and Figure 4, for the torsion angles (ф_1_ and ф_2_) of FL, MAA and 4VPy. Barr = Barrier, MinRel = relative minima and GeomMin = geometries of minima energy, Barr1, Barr2 and Barr3 are maximums in the PES.

Reference	FL´s Conformation	MAA´s Conformation	4VPy Conformation
Initial	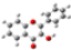	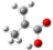	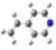
Barr1	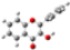	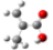	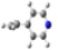
MinRel1	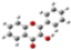	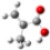	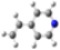
Barr2	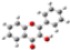	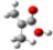	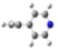
MinRel2	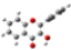	-------	-------
Barr3	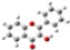	--------	-------
GeomMin	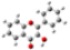	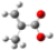	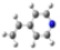

**Figure 3:**
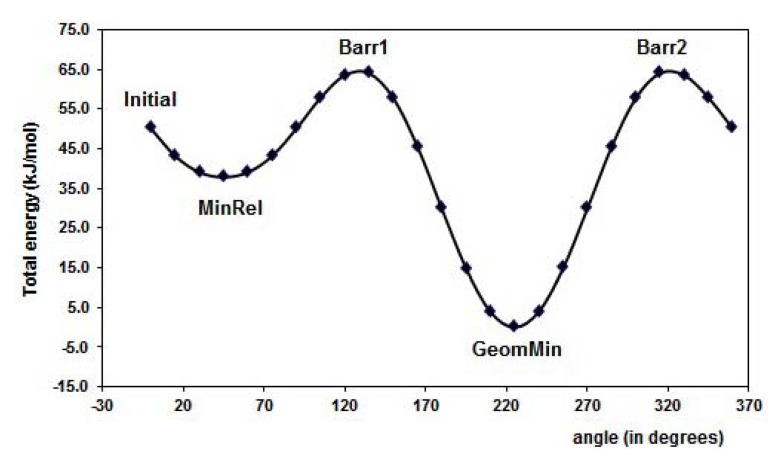
Graphical representation of the PES for methacrylic acid, MAA, for the dihedral angles torsion (ф_1_ and ф_2_), see Figure 1 (b). The corresponding selected geometry structure is in Table 1.

**Figure 4:**
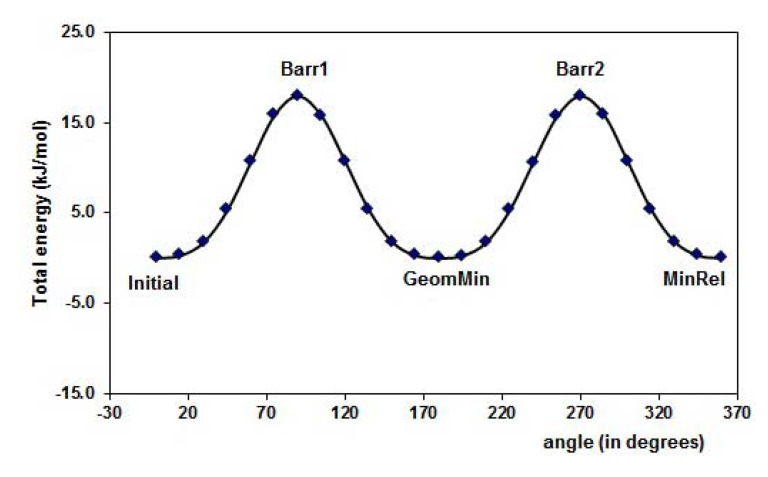
Graphical representation of the PES for 4-vinylpyridine, 4VPy, for the dihedral angles torsion (ф_1_), see Figure 1 (c). The corresponding selected geometry structure is in Table 1.

Figure 3 displays the PES of the MAA for the torsion of the dihedral angle ф_1_, for ф_2_ rotational angle. The minimal energy conformation resulted when the -C=O group is in the plane of the molecule. The hydrogen, H12, is oriented to the outside part of the molecule in the minima energy conformation resulting about 38 kJ mol^-1^ lower than the MinRel1 in which the only difference is the hydrogen orientation in the opposite direction, *i.e.*, pointed out to the inner of the molecule. The different conformations formed along the PES are summarized in Table 1. Furthermore, for the 4-vinylpyridine there is only one dihedral angle ф_1_ and the PES is displayed in the Figure 4. The torsion of this ф_1_ angle gives the planar conformation as the minima energy and the coplanar conformation is the highest value of the PES, about 18 kJ mol^-1^.

The conformational and the Potential Energy Surface (PES) analysis is important not only because it gives the structural form of the molecules with minimal energy, but also because it gives the electronic properties such as atomic charges, possible intermolecular interaction and torsion angles of the isolated molecules, as a reference states, leading to compare differences when a complex between two species is formed. Table 2 displays the ESP and Mulliken atomic charges for the isolated molecules at B3LYP/6-31+G(d,p) level of theory.

**Table 2:** ESP and Mulliken charges distribution for the isolated molecules of flavonol (FL), methacrylic acid (MAA) and 4-vinylpyridine (4VPy), without solvent effect. Labels are referred to Figure 1.

FL				MAA			4VPy				
Atom	ESP	Mulliken	Atom		ESP	Mulliken		Atom	ESP		Mulliken
C1	0.029	0.548	C1		-0.278	-0.574		C1	-0.697		0.420
O2	-0.342	-0.375	C2		0.145	0.412		C2	0.764		-0.007
C3	0.484	-0.371	H3		0.095	0.172		C3	-0.624		0.380
C4	0.111	-0.085	H4		0.091	0.145		C4	0.480		-0.511
**O5**	**-0.557**	**-0.593**	H5		0.095	0.172		**N5**	**-0.721**		**-0.156**
O6	-0.572	-0.580	C6		-0.505	-0.473		C6	0.568		-0.378
C7	0.495	-0.606	C7		0.663	0.417		H7	0.194		0.126
C8	-0.358	-0.085	H8		0.208	0.172		C8	-0.434		-0.368
C9	0.007	-0.267	H9		0.214	0.139		H9	0.198		0.118
C10	-0.243	0.200	O10		-0.598	-0.459		H10	0.029		0.128
C11	0.006	-0.261	**O11**		**-0.559**	**-0.495**		H11	0.007		0.128
C12	-0.244	1.003	**H12**		**0.428**	**0.374**		H12	0.204		0.125
C13	0.333	1.008						C13	-0.289		-0.280
C14	-0.297	-0.455						H14	0.156		0.132
C15	-0.288	0.153						H15	0.164		0.144
C16	-0.101	-0.289									
C17	-0.133	-0.165									
C18	-0.096	-0.448									
H19	0.177	0.140									
H20	0.145	0.136									
**H21**	**0.433**	**0.411**									
H22	0.129	0.128									
H23	0.128	0.130									
H24	0.214	0.158									
H25	0.114	0.137									
H26	0.122	0.165									
H27	0.176	0.135									
H28	0.129	0.128									

Mulliken charge evaluation has more basis set dependence than electrostatic charge (ESP), because the latter is a numerical method whose purpose is to generate charges that reproduce the electrostatic field from the entire wave function [23,24]. This produces charges which are suitable for mechanical calculations, but it can produce meaningless results for atoms which have little exposed surface area [24]. The most important atomic centers considered are highlighted with bold letters. Analyzing and comparing both schemes, ESP charge distribution gives a better description than Mulliken scheme as it is expected. Therefore, from the results in Table 2, it can be deduced that the proton donor in FL is the H21, since it is the most electropositive site and the proton acceptor in this specie is O5 that is the less electropositive site. For MAA the proton donor is H12 and O11 the proton acceptor. Based on these results, it is possible to assume that initial conformation of the complex is formed between FL and MAA on those species. For the case of the 4VPy, there is only one atomic center that can behave as an acceptor site, N5, because its one electron pair can interact with the proton H21 from the FL molecule to form the interaction complex FL-4VPy.

### 2.2. Solvent effect

As it is known, solvent affects meaningfully the formation of the complexes between FL-MAA and FL-4VPy. Therefore, the stability of the FL, MAA and 4VPy in the different solvents, ΔE*_solv_*, namely, chloroform (CHCl_3_), tetrahydrofuran (THF) and acetonitrile (ACN), was calculated to characterize the strength of their respective interaction with them. Where ΔE*_solv_* is defined as:

ΔE_solv_= E_(solution)_ - E_(in vacuum)_ (1)

Then, the ΔE*_solv_* of FL in the three different solvents, CHCl_3_, THF and ACN, was calculated according to Eq. (1) by means of the PCM method implemented in Gaussian 03 package [25]. The results are shown in Table 3. The ΔE*_solv_* of FL in ACN is the largest, indicating the strongest interaction between FL and ACN, while in CHCl_3_ results the smallest and THF had an intermediate interaction strength with FL, as compared with ACN and CHCl_3_. The same calculation procedure was performed for MAA and 4VPy. The results are summarized in Table 4 and Table 5.

**Table 3:** ΔE*_solv_* of FL in different solvents.

Environment	Energy (u. a.)	∆E (kcal mol^-1^)	∆E (kJ mol^-1^)
*In vacuum*	-803.3429098	---------------	----------------
CHCl3	-803.3547000	-7.3983505	- 30.9546985
THF	-803.3565292	-8.5461735	-35.7571899
ACN	-803.3599984	-10.7230965	-44.8654358

**Table 4:** ΔE*_solv_* of MAA in different solvents.

Environment	Energy (u. a.)	∆ *E (*kcal mol^-1^)	∆ *E (*kJ mol^-1^)
*In vacuum*	-306.5084406	---------------	----------------
CHCl_3_	-306.5193031	-6.8162188	-28.5190595
THF	-306.5209396	-7.8431225	-32.8156245
ACN	-306.5237232	-9.5898315	-40.1238550

**Table 5:** ΔE*_solv_* of 4VPy in different solvents.

Environment	Energy (u. a.)	∆ *E (*kcal mol^-1^)	∆ *E (*kJ mol^-1^)
*In vacuum*	-325.709397	---------------	----------------
CHCl_3_	-325.716818	-4.6566775	-19.4835387
THF	-325.718006	-5.4021475	-22.6025851
ACN	-325.720076	-6.7010725	-28.0372873

For these isolated molecules the magnitude of ΔE*_solv_* of MAA and 4VPy in the different solvents shows the same order as FL, *i.e.*, | ΔE*_solv_* (ACN)| > | ΔE*_solv_* (THF)| > | ΔE*_solv_* (CHCl_3_)|. Based on the results in Table 3, Table 4 and Table 5, it is expected that ACN has the highest affinity to the template molecule and the monomers. As it has been mentioned [8], this affinity could acts as a shield or may reduce the interaction between FL and MAA or FL and 4VPy, which is required in the formation of the pre-polymerization complexes. CHCl_3_ has the least effect in the complexes formation, as indicated by its smallest ΔE*_solv_*. Therefore, the MIP synthesized in CHCl_3_ will have a highest selectivity because of the minimal interference of the solvent on the interaction between FL and MAA or 4VPy.

### 2.3. Pre-polymerization: complex formation stage

**Figure 5:**
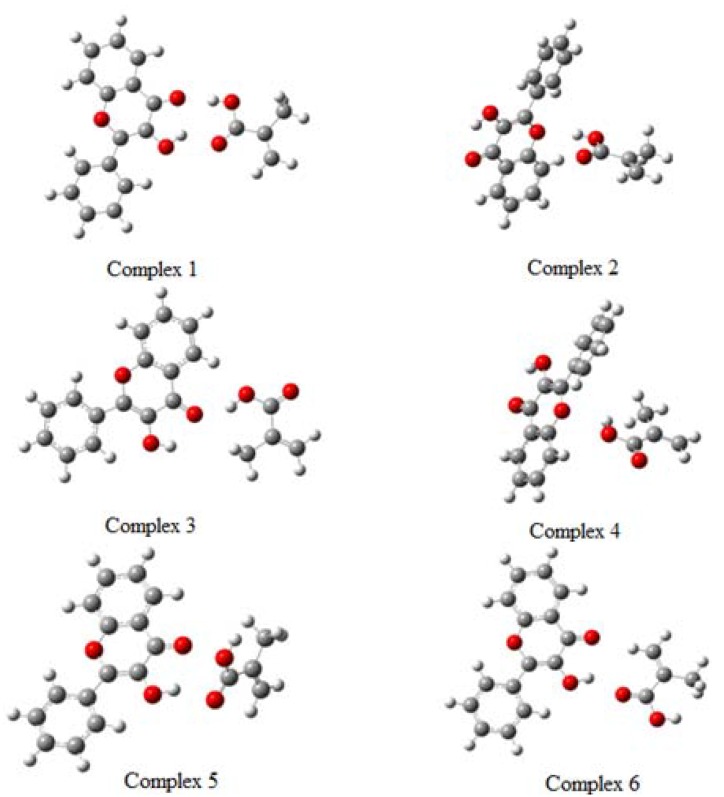
Pre-polymerization complexes proposed in a FL-MAA molecular ratio 1:1.

Based on the PES for every species and also in the nature of the atomic centers charge distributions, different complex conformations were proposed. These complexes were optimized to obtain energy differences among them. Figure 5 shows the optimized geometries of the different proposed complexes, with a 1:1 ratio FL-MAA (complexes 1 to 6).

**Figure 6:**
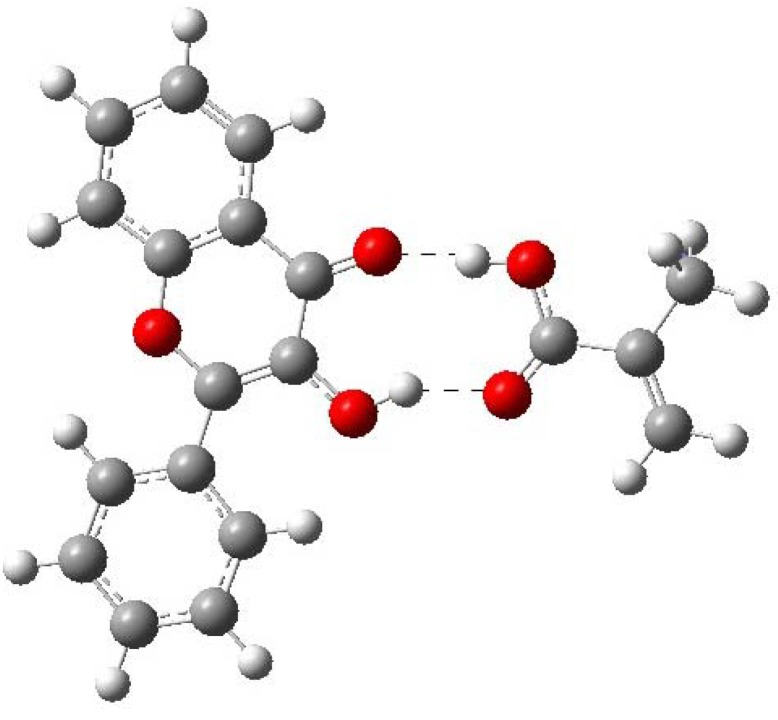
Optimized structure of FL-MAA complex. The Figure shows the two recognitions sites.

Table 6 displays the energy values *∆E* and ∆E* for these different complexes. *∆E* was evaluated from the eq. (2). The negative value indicates a stabilization energy. Hence, the complex 1 was the most stable according to the charge distribution analysis. Then, for the complex 1, there are two atomic centers of the template and two atomic centers of the monomer that give an interaction stronger than the rest of the obtained complexes (see Figure 6). Complexes 2 and 3 are also stabilized in minor degree than complex 1, and the rest of the complexes are not stabilized, (see Table 6). The stabilization energy of the complex 1 is about -12.13 kJ mol^-1^ with respect to the free fragments, complexes 2 and 3 are stabilized by 1.8 kcal kJ mol^-1^ and -1.67 kJ mol^-1^. In the other hand, complex 1 is more stable than complex 2 and 3 in the order of 10.33 and 10.455 kJ mol^-1^, respectively, (see Table 6). The interaction distances in the complex 1 are 1.82 Å for H21-O11 and 1.63 Å for H12-O5. The distance H12-O5 in the complex 2 is 2.1 Å and H12-O5 in the complex 3 is 1.85 Å.

**Table 6:** Binding energies, ∆*E*, between flavonol and MAA and their possible complexes.

Species	Energy in Hartrees	*∆E* (kcal mol^-1^)	*∆E* (kcal mol^-1^)
Flavonol	-803.3429098		
MAA GeomMin	-306.5084406		
MAA MinRel1	-306.4977442		
Complex 1	-1109.870675	-12.13	0.00
Complex 2	-1109.854211	-1.80	10.33
Complex 3	-1109.854015	-1.67	10.45
Complex 4	-1109.845292	3.80	15.93
Complex 5	-1109.846708	2.91	15.04
Complex 6	-1109.847501	2.42	14.54

*∆E* was calculated with the eq. (1), ΔE= E_(complex)_ - E_(FL)_-E_(monomer)_; *∆E*= Complex ***i*** – Complex 1, where ***i*** corresponds to the complex under study

### 2.4. Evaluation of the binding energy

**Figure 7:**
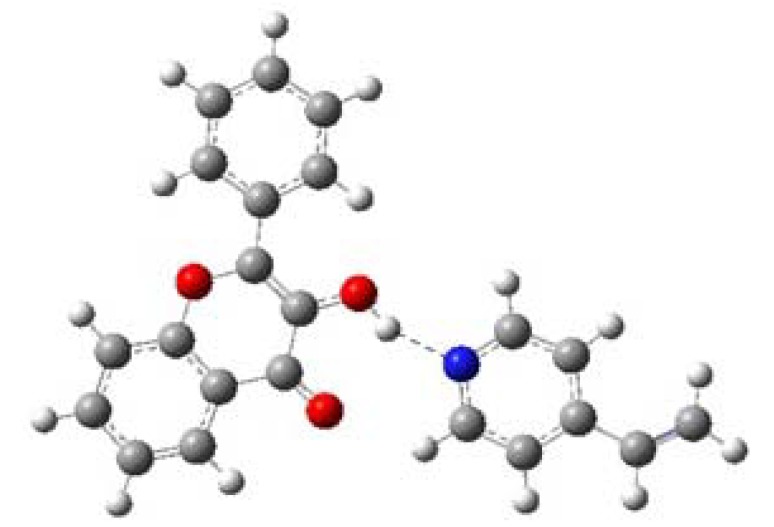
Geometrically optimized structure of FL-4VPy complex. The figure shows one recognition site.

Table 7 shows that ∆*E* of MAA is larger than ∆*E* of 4VPy, indicating that former interaction posses more strength while with 4VPy is rather weak. Hence, the MIP synthesized with MAA is expecting to give the highest selectivity to FL, while the MIP synthesized with 4VPy will give the lowest selectivity. Based on the computed results, it is found that MAA is able to interact as both, hydrogen bond donor (O11-H21) and hydrogen bond acceptor of the FL (H12-O5). On the contrary, 4VPy can only interact with the hydrogen bond donor of the FL (N5-H21). See Figure 6, Figure 7 and Table 7 to compare this.

**Table 7:** Binding energies, ∆*E*,of FL with MAA and 4VPy.

Species	Energy (u. a.)	∆*E (*kcal mol^-1^)	∆*E (*kJ mol^-1^)
FL (free)	-803.3429098	------------	------------
MAA (free)	-306.5084406	------------	------------
4VPy (free)	-325.7093970	------------	------------
Complex 1 (FL-MAA)	-1109.8706749	-12.125559	-50.7333389
Complex (FL-4VPy)	- 1129.0627294	-6.5399305	-27.3630692

*∆E* was calculated with the eq. (1), ΔE= E_(complex)_ - E_(FL)_-E_(monomer)__._

Table 8 shows the electronic stabilization energies in vacuum and stabilization energy in CHCl_3_ for the most stable FL-MAA and FL-4VPy pre-polymerization complexes. According to the computed results when CHCl_3_ is accounted for as a solvent, a minimal effect is obtained. It seems that only in the first stage, before reaction, a stabilization of the free fragments are given by the solvent environment, but once the interaction between the template and monomer takes place, the solvent (CHCl_3_) has no effect in the complex stabilization.

**Table 8:** ΔE*_solv_* of FL-MAA and FL-4VPy complexes in CHCl_3_ solvent. The solvent effect was calculated by PCM.

Species	Energy (u. a.)	∆ *E _solv_ (*kcal mol^-1^)	∆ *E _solv_ (*kJ mol^-1^)
*In vacuum*			
Complex 1 (FL-MAA)	-1109.8706749	------------	------------
Complex (FL-4VPy)	- 1129.0627294	------------	------------
*With solvent effect (CHCl_3_)*			
Complex 1 (FL-MAA)	-1109.86921	-0.9062	-3.79
Complex (FL-4VPy)	- 1129.062447	-0.0003	-0.74

*∆E _solv_* was calculated with the eq. (1), ∆E_solv_= E_(in vacuum)_ - E_(solution)_

### 2.5. ^13^C-NMR spectroscopy

**Figure 8:**
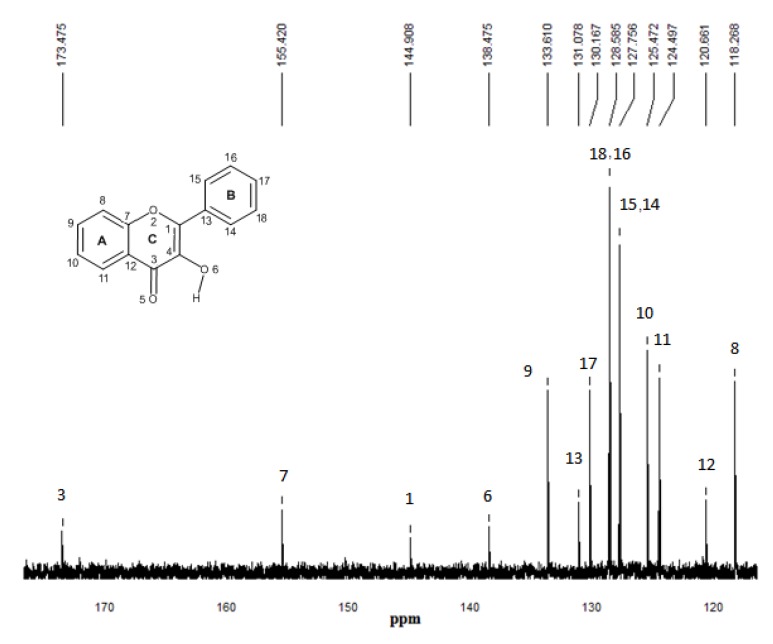
^13^C-NMR experimental spectrum of flavonol in CDCl_3_. The numbering that appears on each signal is relative to carbon atoms.

**Figure 9:**
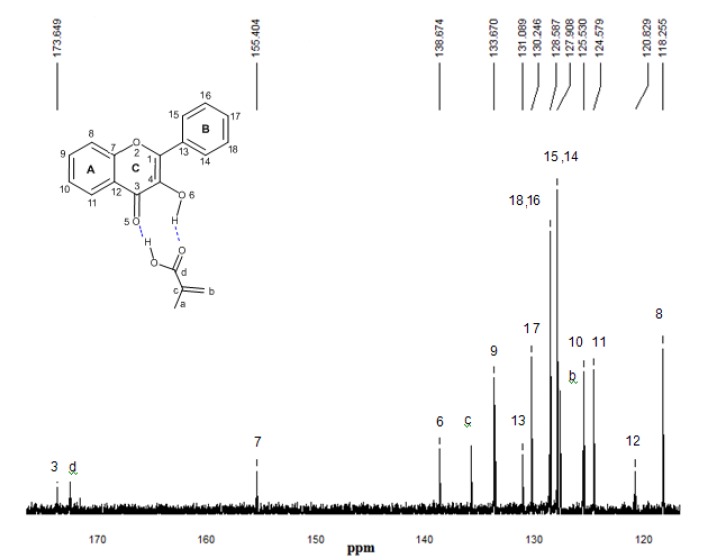
^13^C-NMR experimental spectrum of the complex FL-MAA 1:1 in CDCl_3_. The numbering that appears on each signal is relative to carbon atoms.

**Figure 10:**
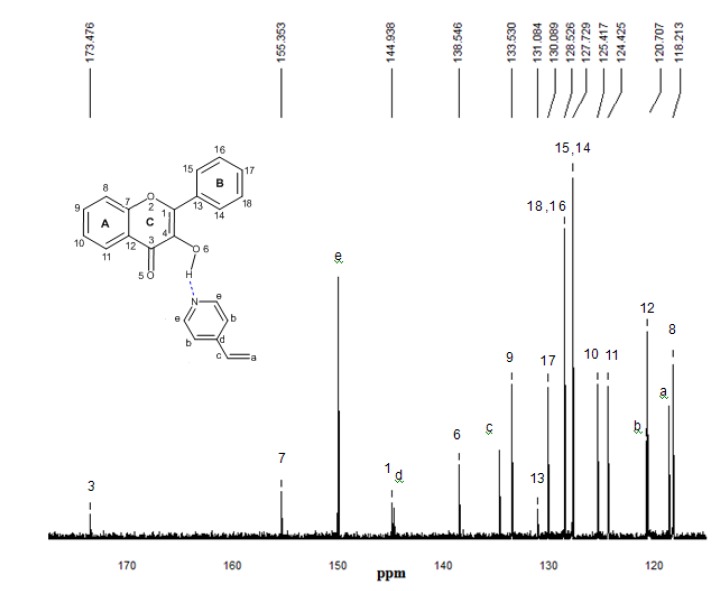
^13^C-NMR experimental spectrum of the complex FL-4VPy 1:1 in CDCl_3_. The numbering that appears on each signal is relative to carbon atoms.

^13^C-NMR experimental spectra for the free flavonol and FL-MAA, FL-4Vpy pre-polymerization complexes were performed to determine the differences in chemical shifts for carbonyl and carbinol groups for that flavonol and flavonol-monomer complex, as it can be seen in the Figure 8, Figure 9 and Figure 10.

^13^C-NMR experimental analyses were used to confirm the presence of interactions between functional monomers and flavonol through the possible displacements. The ^13^C-NMR spectrum of flavonol is shown in Figure 8. The assignments of the flavonol carbons have been made by considering available literature data [26] as it is depicted in the spectrum. From the experimental results, it can be seen that the formation of non-covalent one to one monomer-flavonol interaction affects the resonance of each carbon atom, more specifically the C3 and C4 atomic centers of flavonol, that are directly involved in the interactions of the template and functional monomers. Then, comparing the spectra of FL and FL-MAA complex in Figure 8 and Figure 9, it can observed that C3 and C4 signals were affected by the presence of MAA. During FL-MAA complex formation, the C3 and C4 atoms from FL are deshielded of 0.199 and 0.174 ppm with respect to FL, respectively. Concerning FL- 4VPy complex, the shift of downfield was 0.071 and 0.001 ppm for C3 and C4 atoms of FL. According to the displacement obtained from the ^13^C-NMR experimental evidence, it can assume that those chemical shifts are related with the monomer interaction. Therefore, the FL interaction has more strength with MAA than 4VPy because the slight displacement for C3 atom in the FL-4VPy complex shows that this atomic center remains close to that obtained for FL, see Figure 8 and Figure 10. Hence, no interaction was formed around C4 atom in the FL-4VPy as compared to FL-MAA case. The experimentally observed chemical shifts in these cases are rather small, as it is expected, this is due to the monomers interaction is weak since only van der Waals and hydrogen bonds are present in the molecular interaction. The theoretical ^13^C-NMR was also calculated, the level of calculation for the ^13^C-NMR was HF/6-31G(d) used the GIAO method implemented in *Gaussian 03* [25], taking as a reference for the calculations also TMS. This approach is obtained by computing the absolute chemical shift for TMS and taking the difference between that value and the absolute shift computed for the nucleus in question. The calculated spectra result in the same order of carbon signal positions in agreement with experimental observations, for the FL, FL-MAA and FL-4Vpy. The quantitative comparison between the theoretical deviations from the experimental data ranged about ±5% in average for the main displacements involved in ^13^C-NMR analysis stand for the C3 and C4 atomic centers in FL. Thus, ^13^C-NMR was applied as an index to reflect the magnitude of molecular interaction between FL and the monomers.

The experimental and theoretical results obtained for the ^13^C-NMR spectra demonstrate the effect of the functional monomer-template complex and the availability of computational approach as a useful tool for the understanding of the interactions responsible for complex formation and notwithstanding the ^13^C-NMR spectra provide valuable information, the ^1^H-NMR spectrum would be useful to determine chemical shifts of hydroxyl group proton (position 3 of the γ-pyrone) before and after the interaction of flavonol with each monomer. However, this proton behaves as an acid and should expect a very broad signal between 10–12 ppm. In our experimental conditions, the corresponding proton signal was not observed leading to consider only the possibility of ^13^C-NMR.

## 3. Procedures

### 3.1. Molecular simulation

Study of electronic structure includes all-electrons within the Kohn-Sham implementation of density functional theory (DFT). The level of theory used in this work corresponds to the non-local hybrid functional B3LYP [21,22] whereas the Kohn-Sham orbitals are represented by 6-31+G(d,p) basis set implemented in *Gaussian 03* [25]. The atomic punctual charge was evaluated under the traditional Mulliken scheme and electrostatic charge (ESP) distribution [23,24]. Geometry optimization calculations were carried out for all the involved systems using the Berny algorithm. All the stable structures were further characterized as a minimum in the potential energy surface (PES) by using analytical second derivatives through the frequency calculation. Then, the conformation and the energy calculation were applied to the complex between flavonol (FL) and methacrylic acid (MAA), or 4-vinylpyridine (4VPy), respectively. Therefore, the binding energy of the pre-polymerization complex was obtained from the following equation:

ΔE= E_(complex)_ - E_(FL)_-E_(monomer)_ (2)

The convergence criterion was 10^-6^ Hartrees for the energy, 0.000450 for the Maximum Force and 0.001800 for the Maximum Displacement. In order to consider condensed-phase effects, polarizable continuum model (PCM) [27,28,29] implemented in Gaussian 03 was used. In this model the solvent is represented by a dielectric continuum characterized by its relative static dielectric permittivity єo. The solute, which is placed in a cavity created in the continuum after spending some cavitation energy, polarizes the continuum, which in turn creates an electric field inside the cavity.

### 3.2. ^13^C-NMR

^13^C-NMR was performed to determine the differences in chemical shifts for carbonyl and carbinol groups for that flavonol and flavonol-monomer complex. All spectra were recorded on a Varian Mercury spectrometer operating at 200 MHz. The chemical shifts are in ppm, CDCl_3_ was used solvent and (CH_3_)_4_Si (TMS) was used as reference standard.

## 4. Conclusions

The current quantum-chemical calculations showed an effective evaluation method to elucidate the type and stability of molecular interactions between template and functional monomers during pre-polymerization systems of molecularly imprinted polymers. DFT stabilization energies, ∆*E*, lead to the recognition of molecular complexes and their atoms involved in interaction between template molecule and functional monomer. The binding energy, ∆*E,* obtained with MAA is 6 kcal mol^-1^ lower than that of 4VPy which indicates that flavonol interacts more strongly with MAA than with 4VPy. Therefore, a MIP synthesized with MAA is expected to give the highest selectivity to flavonol. The computational results showed that FL has two recognition sites to MAA functional monomer, while to 4VPy only one recognition site is obtained, in agreement with current ^13^C NMR experimental results. MAA presents a simultaneous double interaction to FL, whereas 4VPy shows a single interaction to FL through its nitrogen atom, although the order of magnitude of each interaction is rather similar in terms of absolute energy.

The solvent has an important role during the polymer synthesis. The theoretical results considering solvent effect showed that chloroform has less affinity to both the template molecule and the monomers allowing the formation of H-bond between the template molecule and monomer. These results show that quantum-chemical calculations, based on DFT method, can be used for screening a library of functional monomers for a specified imprinted molecule.
